# Tobacco and the invention of quitting: a history of gender, excess and will-power

**DOI:** 10.1111/j.1467-9566.2012.01529.x

**Published:** 2013-04-21

**Authors:** Cameron White, John L Oliffe, Joan L Bottorff

**Affiliations:** 1 School of Nursing, University of British ColumbiaVancouver B.C., Canada; 2 >Institute for Healthy Living and Chronic Disease Prevention, University of British Columbia OkanaganKelowna B.C., Canada

**Keywords:** tobacco, gender, will-power, quitting

## Abstract

Since the rise of concern about the relationship between smoking and health in the 1950s and 1960s, the tobacco industry has emphasised notions of individual choice to negate the arguments of the public health sector and legitimatise the industry’s presence in the marketplace. Central to this notion of individual choice has been the idea that the control of tobacco consumption (including quitting) is a function of will-power and that smokers can quit if they really want to. This article examines the way will-power developed as the centrepiece of debates about smoking consumption and cessation in the 1950s and 1960s.

## Introduction

Throughout the second half of the 20^th^ century to the present, the tobacco industry has countered calls for regulatory intervention by maintaining an ideological emphasis on consumer free choice (Balbach *et al.* 2006, Drew 1965). This emphasis on consumer free choice has been accompanied by a vision of the consuming subject as individualised, free-willed and enterprising. One of the most important ideas associated with this vision of the tobacco-consuming subject has been the definition of cessation as a freely available choice. As Philip Morris chief executive officer Michael Szymanczyk put it in June 2000: ‘People who want to quit smoking can quit’ (Henningfield *et al.* 2006: iv27).

This definition of quitting as a choice or a decision rests upon the concept of will-power. The concept of the will, or will-power, is important in this context because, as Mariana Valverde illustrates, it provides a link between the mind and the body and, more specifically, it endows the mind with the capacity to administer and control the body (Valverde 1998). As such, the concept of the will or will-power suggests that we are free to choose what happens to our bodies (Berlant 2007, Rose 1996). Foucault describes this sense of the autonomous self as our ‘relation to ourselves’ (Rose 1996: 26).

Focusing on the 1950s and 1960s, this article examines one aspect of the way that quitting was defined as an act of choice governed by the will. Central to the definition of quitting during this period was the suggestion that it was not smoking *per se* that was dangerous. Rather, the problem lay with excessive smoking. The emphasis on excess enabled the danger of tobacco to be compared with that of other products such as chocolates and fatty foods, which were dangerous when consumed in large amounts (Henningfield *et al.* 2006: iv27, Hirschhorn 2008, Mars and Ling 2008). This argument defined responsibility for consuming and quitting tobacco in terms of the will-power and self-discipline of the individual rather than the addictive qualities of nicotine or the actions of the tobacco industry (Cummings *et al.* 2006).

The cultural and political influence of the association between smoking cessation and will-power was a function of two important factors. The first was its capacity to draw on an Enlightenment narrative about the nature, meaning or ontology of the individual. In other words, the definition of quitting as an act of will-power rested upon the belief in the capacity of the individual to act freely, as Kant (1784) put it in a foundational text on the Enlightenment.

Also intricately connected to free choice and will-power were gender politics that positioned the self-made man’s (Kimmel 1997) embodiment of masculine ideals, including decisiveness and autonomy, as the catalyst for smoking cessation. As many feminist analyses have suggested, the notion of the autonomous individual with the capacity to control itself privileges the male body as the dominant ideal. Rosalyn Diprose suggests that ‘the concept of the individual (as owner of property in one’s person)’ and with an associated capacity for ‘autonomous self-representation’ (or ‘self-presence’) is an ‘implicit male norm’ (Diprose 1994: 50). Moira Gatens similarly states that it is specifically the *male* subject which has been ‘constructed as self-contained and as an owner of his person and his capacities’ (Gatens 1991: 5). These links to gender are important because they help to explain the influential role that the notion of will-power has played in conceptions of quitting, at both a political and cultural level. The argument that will-power needs to be understood as a masculine ideal suggested that the only thing that men needed to do to manage their cigarette consumption was, quite simply, to be men (White *et al.* 2012). At the same time, the definition of tobacco reduction or smoking cessation as an expression of masculine virtue (that is, will-power and self-control) was also important because it anchored the inability to quit as unmasculine and effeminate, residing with gendered representations of women as irrational and that were also embodied by weak men (Cieply 2010, Roberts 1998). These gendered politics prioritised individual responsibility and militated against a more rigorous approach to the regulation of the tobacco industry (Bayer and Colgrove 2004, Brandt 1990).

The public health community has long sought to counter the tobacco industry’s definition of cessation as an act of free choice. The most important aspect of this public health campaign has been an emphasis on the addictive qualities of nicotine (Rasmussen 2010, US Department of Health, Education, and Welfare 1988). Addiction suggests that consumption is not a free choice, that desire cannot be controlled by the will and that quitting is extremely difficult, or indeed impossible, for many consumers (Henningfield *et al.* 2006). In other words, as Michael Warner says, ‘desire and will become distinct’ (Warner 1996: 32). The significance of the current article lies in the way that it provides a historical and theoretical framework within which public health can consider and engage with the ongoing capacity of the free will to inhabit notions of tobacco cessation (Redfield and Brodie 2002).

## Excessive consumption

In the course of work investigating the relationship between smoking and health, the scientific and popular literature during the mid-20^th^ century defined the dangers of tobacco in terms of excessive consumption. This emphasis on excess was widespread at both a popular and scientific level and was evident in some of the most formative tobacco documents of the mid-twentieth century. A 1950 article by Wynder and Graham for the American Cancer Society, published in the *Journal of the American Medical Association*, stated: ‘*Excessive* and prolonged use of tobacco, especially cigarettes, seems to be an important factor in the induction of bronchogenic carcinoma’ (Wynder and Graham 1950: 8, my italics). William Leib, in an equally ground-breaking article published in the *Readers Digest* in 1953, argued: ‘Used to *excess*, tobacco may be the cause – sometimes unsuspected – of grave physical ills’ (Leib 1953: 45, my italics).

In June 1956, when the US Public Health Service formed a scientific study group on the subject of smoking and health, the group concluded, similarly: ‘there is a causal relationship between *excessive* smoking of cigarettes and lung cancer’ (US Department of Health, Education and Welfare 1964). During hearings held into cigarette advertising during the first half of 1957, Surgeon General Leroy E. Burney testified: ‘It is clear there is an increasing and consistent body of evidence that *excessive* cigarette smoking is one of the causative factors in lung cancer’ (Federal Trade Commission [FTC], 1964: [10], my italics). Burney repeated this emphasis on excessive smoking in a statement issued on 12 July 1957 (Burney 1957). The emphasis on excessive smoking continued throughout the next decade. In 1959 the American Public Health Association called for action on tobacco because ‘scientific evidence has established that *excessive* cigarette smoking is a major factor [in lung cancer]’ (FTC 1964: 11, my italics).

## The distinction between excess and moderation

Excess is, by definition, a relative term. Instead of emphasising abstinence, according to which all use could be construed as abuse, the opposite of excess was constructed as moderation. The emphasis on excess was significant, in this respect, because it made it possible to construe moderate tobacco consumption as itself an indication of self-control and personal autonomy. As Diprose writes, citing Foucault, ‘moderation has an essentially masculine structure of active virility, ‘immoderation derives from a passivity that relates it to femininity and hence women’ (Diprose 1994: 30):

The significance of moderation was emphasised in a number of different environments. Mr Blatnik, the head of 1957 Congress Commerce Committee hearings into the role of cigarette filters in advertising, argued:

It is not the use of a bad thing, if you want to say cigarettes are bad. It may not be so much the use of a bad thing as the abuse of a good thing … It has to be done in moderation. (Blatnik *et al.* 1957)

The commentary about the cigarette controversy that emerged in the context of the Surgeon General’s 1964 report was also replete with examples of the valorisation of moderation. The *World Telegram and Sun*, for example, published its response to the Surgeon General’s report in an article entitled ‘Moderation is golden’. Whether the article was right or wrong was, perhaps, less significant than the fact that it was expressing an opinion in a public forum. ‘The [Surgeon General’s] Committee’, the article stated, ‘dwells on moderation’ (*World Telegram and Sun* 1964). The *Sun*, from Springfield Ohio, argued: ‘There is no evidence, and no significant statistical implication, that *moderate* cigarette smoking is “dangerous to health”,’ (*Sun* 1964, my italics). This argument was reiterated in 1969 by the Governor of Virginia, Mills E. Godwin Jr: ‘Cigarette smoking … in moderation’, he said, ‘has not been conclusively proved to be injurious to your health’ (Godwin 1969: 628).

The normalisation of moderate tobacco consumption was made all the more significant by the fact that it was difficult, if not impossible, to actually define what moderate tobacco use actually looked like. This was partly a function of the fact that many in the public health sector argued that there was no such thing as moderate consumption and that any and all consumption was dangerous. Thus, amid an inability to agree on even the criteria by which moderation might be evaluated (Harris 2005), it appeared that the best and only way of determining what moderation was, was by comparing it with what it most clearly wasn’t. Thus it almost seemed as if smoking could be construed as moderate up until the point of illness. One tobacco industry document, for example, suggested a distinction between ‘heavy smoking’ and ‘excessive smoking’ (Heimann and Cohen 1962: 18). ‘Excess’, in other words, was beyond ‘heavy’. This situation, from the point of view of the tobacco industry, was nearly perfect.

## Defining the tendency to excess

The distinction between excess and moderation raised the question: what qualities inclined some smokers to excess and not others? In the words of one tobacco industry document, what inclined certain individuals to ‘excess-proneness’ (Heimann and Cohen 1962)? Some argued that individual susceptibility to excess tobacco consumption was a question of constitution. This manifested itself in the so-called ‘constitutional hypothesis’ (Gundle *et al.* 2010: 975). As described by Dr Leo Katz, Professor of Statistics at Michigan State University, the constitutional hypothesis, ‘treats the individual as almost unique among all possible individuals’ (Katz 1969: 863). Dr Zeidman, Professor of Pathology at the University of Pennsylvania, stated: ‘Those who are able to give up smoking may be constitutionally or humorally different’ (Zeidman 1969: 1050). The ‘constitutional hypothesis’ continued to be espoused, particularly by the tobacco industry, throughout the 1970s (Lincoln and Morris, 1972) and eventually evolved, as Gundle *et al.* illustrate, into research into the ‘genetics of nicotine addiction’ (Gundle *et al.* 2010: 974).

While many argued that individual susceptibility to excessive tobacco consumption was a question of constitution, the idea that the individual capacity to manage the effects of tobacco needed to be understood in terms of psychology and personality types was also influential. Perhaps drawing on earlier, 1930s, psychological studies on narcotics, which had defined narcotic addiction as a function of a pathological character (Acker 2002, Campbell 2007), research including the ‘Psychology of smoking’ by Charles McArthur *et al.* dated 1953, for example, framed the question of who can stop smoking in terms of psychiatric differences. Those who can stop were labelled as having a strong basic personality, and being sociable and practical. Those who can’t stop were labelled as having a weak basic personality, being asocial with a lack of purpose and values, and introspective, ideational and inhibited (McArthur *et al.* 1953).

A 1958 study by Clark Heath, entitled ‘Differences between smokers and non-smokers’ categorised men’s smoking habits in terms of similar psychiatric qualities and personality traits. The findings of the study, Heath argued, illustrated that differences between men who smoked heavily and men who smoked moderately or not at all could be understood in terms of personality. Heath concluded that the basic difference between male smokers and non-smokers was on their degree of dependability and self-control. He suggested that the heavy smokers showed symptoms of being impulsive and volatile. They were also restless and inclined to seek danger. They also exhibited a kind of independence, which created difficulties with marriage. Non-smokers, Heath argued, had different personalities. Although ‘somewhat on the bland, colorless side’, non-smokers nevertheless possessed ‘the more stable qualities of dependability and good direction of aims in life’. They were ‘steady, dependable and hard working with stable marriages’ (Heath 1958: 379–80).

Clarence Cook-Little, Scientific Director of the Tobacco Industry Research Committee, rehearsed a similar argument. He also explained a tendency towards excess in terms of psychiatric qualities and personality traits. Cook, who had a very visible public presence during the late 1950s and was widely cited in newspaper reports, associated heavy smoking with certain differences, imbalances, abnormalities or non-adjustments. These differences or imbalances were defined variously as ideological, psychological, emotional, physio-psychological, or neuro-hormonal. They created what Cook-Little described as an ‘off the normal’ individual marked by a ‘lack of balance’, ‘nervousness’, ‘restlessness’, or some other ‘nonadjustment to some phase or phases of life’ (*New York Post* 1957).

## Will-power hypothesis

The findings of research into the personalities of male smokers led to the suggestion that an inability to control consumption, leading to excess, was a function of restlessness, a lack of purpose, impulsiveness, volatility and so on. These qualities were also associated with a lack of self-discipline and self-control. Excessive smoking, in other words, became defined as a function neither of tobacco itself nor even of any innate or physiological, psychological or psychiatric qualities in individuals. Rather, excess came to be associated with the pseudo-scientific, pseudo-philosophical concept of will-power. The significance of this emphasis on will-power was that it suggested that male smokers who smoked excessively could flex their will and stop their destructive behaviour if they really wanted to. Hence any and all health repercussions emanating from men’s smoking became a personal responsibility.

The emphasis on the idea that tobacco control could and should be based on will-power and self-control was evident in a range of documents produced during the mid-20^th^ century. A 1962 internal tobacco industry document entitled ‘Heavy smokers with low mortality and excess-proneness with high mortality’, for example, written by American Tobacco Company President R. K. Heimann, suggested that some individuals were ‘excess prone’ on account of ‘urban factors’ (Heimann and Cohen 1962: 18). These included ‘greater anxiety, greater psychological tension and *less individual restraint*’. Reiterating earlier studies cited above, this document argued that excessive tobacco consumption needed to be understood in the context of other phenomena related to a man’s lack of restraint. These included a greater tendency towards divorce as well as tendencies to ‘work more, to drink more, [and] perhaps to eat more’ (Heimann and Cohen 1962: 10, my italics).

An emphasis on will-power and self-control proliferated in the aftermath of the release of the 1964 Surgeon General’s report (US Department of Health, 1964). The *World Telegram and Sun* notes: ‘The [Surgeon General’s] Committee recommends ‘‘firm mental resolve” as the surest way to forsake the habit’ (*World Telegram and Sun* 1964). Dr Joshua Harold Burn, Professor in Pharmacology to Washington University, St Louis Mo, argued, in 1969: ‘There is a great difference between individuals … in the amount of *self-control* they possess in avoiding excesses which lead to ill-health’. This emphasis on self-control was the basic difference, Burn suggested, between ‘the non-smokers’ and ‘the heavy smokers’ (Burn 1969: 1133, my italics). Even Luther L. Terry, former Surgeon General and the architect of the 1964 report on smoking and health, suggested that the capacity to manage the relationship between the self and cigarettes was a function of men’s ‘resistance and self-control’ (Terry 1969: 278).

## The historical gendered significance of will-power

The definition of smoking and smoking cessation as a function of will-power, control over the self (self-control) and individual agency resonated in a number of ways. The significance of this argument was partly a function of the fact that it was so embedded in American culture and history. Allan Brandt describes this argument as a ‘cultural phenomenon’ related to ‘the traditional American libertarian ethic: ‘It’s my body and I’ll do with it as I please’’ (Brandt 1990: 167).

American libertarianism, in turn, is itself part of a political tradition. It can be traced back to the philosophy of John Locke, in whose work individuals are invested with the capacity to own their person, their body, as property. It is in the context of this sovereign relation between the self and the body that the individual is said to be autonomous (Locke 1967). Locke, in turn, was followed by John Stuart Mill who, in *On Liberty* (1859), defined the individual by the dictum: ‘over himself, over his own body and mind, the individual is sovereign’ (Diprose 1989: 29).

The idea that tobacco consumption could and should be managed in terms of the power of the self over the self also resonated, particularly among American men, by virtue of its appeal to the ideal of control over the self as an exemplary sign of masculine virtue. The significance of self-control in the culture of American masculinity can be understood in terms of the ideal of the self-made man. This can be traced back, as E. Anthony Rotundo illustrates, to the late 18^th^ century. This ideal, he suggests, emerged as part of a broader series of changes: the birth of republican government, the spread of a market economy and the concomitant growth of the middle-class itself. ‘The old male passion of defiance’, Rotundo writes, ‘was transformed into the modern virtue of independence’ (Rotundo 1993: 3). De Tocqueville famously described the American male as ‘restless in the midst of abundance’ on account of his earnest pursuit of prosperity and independence (de Tocqueville 1838).

The term itself (self-made) was first used in 1832, Osgerby suggests, by Henry Clay in a Senate speech venerating the ‘self made’ success of Kentuckian entrepreneurs (Osgerby 2001). It quickly caught on, Osgerby argues, as ‘a descriptor of masculine achievement through individual energy and diligence’ (Cawelti 1965, Decker 1997, Osgerby 2001: 19, Wyllie 1954). It was represented in mid-century popular biographies such as John Frost’s *Self Made Men in America* (1848), by Horatio Alger’s ‘pluck and luck’ novels and by the launch, in the late 19^th^ century, of uplifting self-help magazines such as *Success* (1897) and *World’s Work* (1900) (Osgerby 2001). One of the great exponents of the ideal of the self-made man, a man whose future was limited only by his will-power, was Theodore Roosevelt. ‘A man,’ he wrote in his 1913 autobiography:

must by custom and repeated exercise of self-mastery, get his nerves thoroughly under control … [H]is will [power] will grow stronger and stronger with each exercise of it. (Roosevelt 1913: 24).

Finally, the idea that tobacco consumption could and should be managed in terms of capacity of the self to control the self (that is, will-power) also resonated because it drew specifically on already existing discourses and debates about tobacco consumption. John Harvey Kellogg writing in 1922, for example, had argued that ‘the best way to stop smoking is To STOP’. This stopping was a function of determination, heroic perseverance and steadfast decision-making. It was no more or less than a ‘test of character’ (Kellogg 1922: 151). ‘[A person] must organize and perseveringly maintain a systematic and unrelenting campaign,’ Kellogg wrote. ‘He must say, with the heroic Patrick Henry, “Give me liberty, or give me death”’ (Kellogg 1922: 153). Mac Levy, writing slightly earlier than Kellogg, also suggested an emphasis on character as the basis for controlling tobacco consumption. ‘This book’, he wrote in the forward to *Tobacco Habit Easily Conquered*, ‘teaches self-mastery and health regeneration by that much misunderstood, greatly maligned but supremely important personage YOURSELF’ (Levy 1916: 15).

## Implications of the will-power hypothesis

The emphasis on will-power, control over the self (self-control) and individual agency was significant because it employed notions of autonomy or freedom (that is, the autonomy of the self over the self) as the fundamental basis of tobacco control. Freedom, in other words, became the primary program of governance (Valverde 1998). As Nikolas Rose writes: ‘“Free” individuals are enjoined to govern themselves as subjects simultaneously of liberty and of responsibility’ (Rose 1996: 12).

The significance of this emphasis on autonomy and control was that tobacco consumption came to be associated with a particular kind of subject and a particular kind of ideology, an ideology that suggested that the capacities of the individual could be optimised in a free market. In other words, the regulation of tobacco could now be represented as an attack on freedom, an attack on the very idea of the capacity of a man to self-manage.

Some, for example, represented attempts to regulate the tobacco industry as an attack on the right to free choice. Terry Sanford, Governor of North Carolina, put it simply: ‘These people [i.e., smokers] … are consumers by their own free choice’ (Sanford 1964: 464). Dr William J. E. Crissy, Professor of Marketing at Michigan State University, suggested that ‘restrict[ing] or limit[ing] cigarette advertising’ would, cost ‘a very high price … the erosion of consumer freedom and the free market economy’ (Crissy 1969: 1333). Dr Vernon Fryburger, Professor of Advertising and Marketing, Northwestern University, argued that any attempt to regulate the ‘free and voluntary’ consumer response to advertising or goods would, in effect, regulate ‘a powerful electorate, a powerful audience and a powerful consumer’ (Fryburger 1969: 1338).

Yet, as the Fryburger statement illustrates, with its invocation of a powerful electorate, tobacco regulation could be understood not only as a challenge to consumer freedom but as a challenge to political freedom. Thus Dwight Emery Harken, Clinical Professor of Surgery at the Harvard Medical School, argued that the issue at stake in the debate about the regulation of tobacco advertising was no less than individual liberty. He told a 1969 Congress Commerce Committee that while the decision to smoke should be well informed, ‘our concepts of individual liberty are such that everyone should have a free choice in deciding whether or not to smoke’ (Harken 1969: 1392). The National Newspaper Association wondered similarly:

What has happened to the traditional American concept of freedom: freedom of the entrepreneur to present his wares; freedom of the public to hear the advertising messages of all who purvey goods and services? (National Newspaper Association 1969)

## The normalisation of tobacco

The emphasis on the autonomy of the self over the self as the best way to manage the problem of excessive tobacco consumption was also significant because it normalised (as opposed to medicalised) tobacco. It enabled tobacco to be compared with other products for which excessive or indiscriminate consumption could be construed as dangerous but for which normal or moderate consumption was quite safe. This included virtually any other product on the market, including foodstuffs and liquor. Moreover, tobacco could be differentiated from products (primarily illicit drugs) that were considered addictive and deleterious to health *per se.*

By focusing on the issue of self-control as the site at which tobacco became dangerous (or not), many commentators sought to compare tobacco with other substances that were normally benign yet that could cause health problems if consumed in great quantities. Some, for example, compared tobacco with ‘rich foods’ that could lead to a build up of fat and thereby cause heart trouble. Examples of rich foods included candy and butter, cakes and chocolate (Henningfield *et al.* 2006), fatty foods (Cummings, *et al.* 2006), eggs and milk, and cocoa, tea and coffee (*Journal of Commerce* 1964). The *Journal American* from New York suggested that excessive smoking is ‘bad for a person’ in the same way as ‘the … excessive use of anything … Eating too many hot dogs will give you heartburn’ (Gould 1964). Clarence Little of the Tobacco Industry Research Council compared the concern over tobacco consumption with the relationship between sunbathing and skin cancer (Little 1957). Terry Sanford, Governor of the State of North Carolina, compared the dangers of excessive smoking with the dangers of ‘excessive eating, excessive drinking, and excessive speed in automobiles’ (US House of Representatives 1964: 7).

The comparison of the dangers of excessive tobacco consumption with the excessive consumption of other products and experiences, from food to automobile speed, was also applied to the question of the kinds of regulation that constituted an appropriate response to the dangers of tobacco. John C. Williamson, President of the Flue-Cured Tobacco Growers Association in Raleigh, North Carolina, asked:

Who would even suggest that it be prescribed by law how much and what kind of food a person might eat? … [Who would] advocate the abolition of the automobile industry? (US House of Representatives 1964: 64)

The *Times Leader* from Pennsylvania described the 1964 FTC proposal, that all tobacco packaging and advertising should be required to carry a health warning, as the equivalent of suggesting that the consumers of numerous other commodities should be similarly forewarned. If the ‘reasoning of the Federal Trade Commission’ triumphed, and ‘the Commission took over the responsibilities of the medical profession’, the article stated, ‘chaos would prevail’:

Food would be a natural target because … it might be construed as detrimental to health and to life itself. Candy, cakes, bread, canned fruits etc. could lead to diabetes. Meats might affect certain individuals adversely, just as excessive use of other edibles might lead to serious complications to the stomach, heart, or other organs. The list is endless … Since automobiles, planes, railroad trains, buses and trucks kill thousands and injure millions annually, firms would have to include in every appeal for patronage a notice that a journey could wind up in the morgue or hospital. This may sound silly, but it is real under the cigarette ruling.

(*Times Leader* 1964)

The *Post & Times Star* from Cincinnati, Ohio, took the argument to its logical conclusion. Here it was suggested that:

The only logical extension of the FTC’s order is for the soothsayers in Washington to order every newborn American infant be indelibly tattooed as follows: ‘Young man (or woman) you are alive, which is the most dangerous enterprise ever discovered by man. The fatality rate, which is 100 per cent, makes living an extremely hazardous occupation, and one which your government hereby warns you against’. (*Post & Times Star* 1964)

While the dangers of tobacco consumption were compared with the dangers of driving, the dangers of eating and even the dangers of life itself, by far the most common comparison was between tobacco and alcohol (liquor). The *Chicago Daily News* reasoned: ‘Should we require that liquor be branded a health menace because in too-great quantity it can cause kidney and liver disease?’ (*Chicago Daily News* 1964).

The *Post & Times Star,* from Cincinnati, Ohio asked: ‘Why isn’t the FTC entitled to paste a skull and crossbones on every jug of whisky offered for sale?’ (*Post & Times Star* 1964). Others invoked alcohol as an example of how to regulate tobacco, especially tobacco advertising. Brock Adams, a member of the 1969 Committee on Interstate Commerce and Foreign Affairs investigating the issue of cigarette labelling and advertising, suggested:

The television industry could eliminate cigarette advertising as basically it has done hard liquor, and in the [same] times that are involved, which are basically prime times and prime programs, [and] substitute other advertising for it that people are standing in line for. (Henderson 1969: 398)

The comparison between tobacco and alcohol was used to attack the anti-tobacco campaign more generally. James T. Broyhill, a Congressman from North Carolina, accused Mr. Hyde, the Chairman of the 1969 Committee on Interstate Commerce and Foreign Affairs investigating the issue of cigarette labelling and advertising, of attacking the tobacco industry (as opposed to the liquor industry) because it was an easy target, and effectively complained of prejudice against the tobacco producing states:

Here, [in America], cigarettes are produced in only three States, and yet you have got alcohol, hard liquor, and the beer and wine industry all over the United States. Perhaps you are not willing to do battle with them, but you are willing to do battle with these three southern or border States. (Broyhill 1969: 398)

## Discussion and conclusion

In this article the argument has been put forward that the concept of excess helped to create a discursive framework within which tobacco cessation was defined in terms of will-power and self-control. The gendered framing of cessation also suggested that virtuous men might more ably control their consumption if they really wanted to. This enabled a comparison between tobacco and other products which were dangerous if consumed in large quantities but safe when consumed in moderation. This argument subsequently played an important role in the tobacco industry’s defence in court cases throughout the late 20^th^ century. Here, the tobacco industry defined consumers as smart, forceful, determined and independent (Brandt 1990). Related to consumer power, legitimate opportunities were also afforded to many men to demonstrate their estrangement from self-health through discrete practices or a combination of smoking, high fat or high-calorie diets or alcohol overuse. By appealing to men’s will-power men were also freed to define excess on their own terms and in relation to masculine ideals that have long positioned men as more likely to risk than promote their health (Courtenay 2000).

A significant aspect of the emphasis on will-power and self-control was its positioning as most likely to promote smoking cessation and therefore men’s self-health. Drawing on the myth of the self-made man as well as earlier Enlightenment theories of individual agency, the discourse of self-control seemed to suggest that all men could exercise their control and choice to quit smoking. At the same time, an inability to simply quit when one’s mind was made up revealed failed quitters as weak, harbouring a mind–body disconnect that saw them unable to action their will to be smoke-free. This gendered framing has been further invoked by feminist histories of tobacco control that have highlighted the extent to which women’s smoking has been defined as more problematic and as requiring greater public health scrutiny than men’s smoking (Berridge 2001, Oaks 2000). It might even be said that by framing tobacco control in terms of the masculine virtue of self-control and thereby feminising (or emasculating) smokers unable to quit by their own volition, the tobacco industry profited from the generation of that gendered difference (Diprose 1989).

Today, it might seem that the problem of excess has been superseded by the concept of addiction and that the debate about will-power is no longer as significant as it once was. As Paul Knopick, a senior employee at the Tobacco Institute, stated in 1980, ‘we can’t defend continued smoking as a “free choice” if the person was addicted’ (Mars and Ling 2008: 1797). Despite the suggestion that addiction displaces the relationship between cessation and will-power, this is not always the case. Addiction, as Mars and Ling illustrate, is a ‘malleable concept situated in specific social, political, and scientific contexts’ (2008: 1800). This is to say that, under the regime of addiction, the will has continued to be conceived of as the principal site at which consumption can and should best be managed. Quitting has continued to be understood as a function of will-power (Mars and Ling 2008). Balbach *et al.* 2006, for example, argue that dominant tobacco control strategies such as increasing taxes, smoking restrictions and public awareness campaigns reiterate the idea of the ‘individual, rational decision maker’ (Balbach *et al.* 2006 iv37). Chapman and MacKenzie highlight the ongoing influence of notions of individual agency on conceptions of quitting (despite decades of debate about addiction) when they emphasise that most smokers quit unaided, using cold turkey and reducing-then-quitting approaches (2010).

The ongoing focus on will-power despite (or because of) the discourse of addiction has also been addressed by a number of theorists working in the field of addiction more generally. Redfield and Brodie (2002), for example, argue that the discourse of addiction reproduces or, more specifically, ‘inhabits’ the idea of the free will. Sedgwick describes addiction as a ‘counterstructure’ to the concept of the free will, ‘always internal to it, always requiring to be ejected from it’ (Sedgwick 1993: 133–4). This is similar to Valverde’s argument that the representation of alcoholism as a disease (an addiction) has been accompanied by a re-investment in the importance of purposeful and strenuous actions by the patient (Valverde 1998). Hence the language of addiction has continued to depend upon and reiterate a model of the subject that is autonomous, rational, enterprising and responsible (Seear and Fraser 2010).

In the context of the ongoing influence of will-power on contemporary concepts of quitting, recent work by Chapman and MacKenzie (2010) has argued that tobacco control needs to reconsider its view of unassisted cessation. In short, Chapman and MacKenzie suggest that tobacco control’s prevalent view of unassisted cessation as ‘the enemy’ has contributed a widespread focus on pharmacological and designated social supports for quitting. This emphasis on cessation aids, Chapman and MacKenzie suggest, has had the unintended consequence of ‘disempower[ing] smokers’, ‘[eroding] human agency’ and ‘inhibit[ing] quit attempts through anticipatory, self-defeating fatalism’. Chapman and MacKenzie argue that the ‘persistence of unassisted cessation as the most common way that most smokers have succeeded in quitting’ needs to be ‘openly embraced’ by the public health community as ‘an unequivocally positive message’ (Chapman and MacKenzie 2010: 5). With this in mind there might also be some traction for endorsing cold turkey quitting as an effective male-centred tactic.

The historical analysis of mid-20^th^ century thinking about the excessive use of tobacco and will-power in this article makes an important contribution to this contemporary debate about the nature and culture of quitting. This historical analysis suggests the need for these contemporary discussions to consider the way an emphasis on will-power as the basis of quitting defines smoking as the responsibility of the individual consumer rather than the responsibility the tobacco industry. An emphasis on will-power therefore lends itself to the political and legal strategies that have been employed by the tobacco industry to delimit regulation (Balbach *et al.* 2006).

In conclusion, a tobacco control focus on individual agency and will-power such as that proposed by Chapman and MacKenzie (2010) represents a provocative and potentially significant contribution to contemporary tobacco control debates. At the same time, however, additional sociological analyses of tobacco consumption and cessation continue to require consideration. It remains important to continue to understand individual smoking behaviour within the context of a social environment (Poland *et al.* 2006). As Thompson *et al*. (2007, 2009) argue, public health strategies that ignore sociological considerations and instead focus only on the responsibility of the individual can reinforce the emergence of smoking as a marker of socioeconomic difference.
